# The effect of digital media on college students’ sports participation: The mediating role of sports cognition and the moderating role of self-efficacy

**DOI:** 10.1371/journal.pone.0330445

**Published:** 2025-09-05

**Authors:** Yuanji Zhong, Wenhao Guo, Yang Xue, Pengwei Chen, Yongshun Wang

**Affiliations:** 1 School of Physical Education and Arts, Jiangxi University of Science and Technology, Ganzhou, Jiangxi, China; 2 School of Recreational Sports and Tourism, Beijing Sport University, Beijing, China; 3 College of Physical Education, Huaqiao University, Quanzhou, Fujian, China; University of Tartu, ESTONIA

## Abstract

**Background:**

With the increasing integration of digital media into daily life, its influence on college students’ sport-related behaviors has become a growing area of interest. While prior research highlights the general benefits of media exposure, the specific psychological mechanisms through which digital media use affects sports participation remain insufficiently understood.

**Methods:**

Drawing on Social Cognitive Theory (SCT), this study explores how digital media use influences sports participation through the mediating role of sports cognition and the moderating role of self-efficacy. A total of 628 college students (M = 20.14, SD = 1.78) from seven comprehensive universities across China participated in a cross-sectional survey. Data were analyzed using multiple regression analysis and moderated mediation analysis.

**Results:**

Digital media use was positively associated with students’ sports participation. Sports cognition significantly mediated this relationship, suggesting that media use enhances participation indirectly by shaping individuals’ knowledge and understanding of sports. Additionally, self-efficacy moderated the link between cognition and behavior, indicating that individuals with higher self-efficacy are more likely to convert sports-related knowledge into action.

**Conclusion:**

These findings contribute to a more nuanced understanding of how digital media engagement supports sports participation through both cognitive and motivational pathways. The results offer practical implications for educators and media developers seeking to foster physical activity among college students by leveraging digital platforms.

## 1. Introduction

Sports participation, defined as the voluntary involvement in physical activities and the associated decision-making processes [1], is widely recognized as a fundamental contributor to individual well-being. The World Health Organization’s Global Status Report on Physical Activity 2022 underscores that regular participation in physical activity contributes significantly to both physical and psychological health. Despite this well-established understanding, a substantial proportion of the global population fails to meet minimum activity standards. Alarmingly, over 80% of adolescents and 27% of adults worldwide remain physically inactive relative to WHO guidelines [2]. In the Chinese context, a similar pattern of inactivity is evident among university students. Although national policies have increasingly emphasized the importance of campus-based fitness and sport participation, empirical data suggest limited behavioral change. Nearly half of the college student population reports engaging in exercise less than three times per week, with individual sessions frequently lasting under 30 minutes [3]. This insufficient level of participation not only undermines physical fitness but also poses risks to students’ emotional regulation, stress management, and social functioning [4].

Extensive literature has documented the physiological and psychological benefits of sport involvement, such as enhanced motivation, improved academic performance, and a higher overall quality of life [5]. Nevertheless, existing research has predominantly concentrated on adolescent populations or elite athletes [6], with comparatively little attention given to college students. This group, currently undergoing a formative period characterized by evolving identities and increasing autonomy, exhibits unique behavioral patterns that remain insufficiently explored. At the same time, the digital transformation of student life has introduced both promising avenues and complex challenges for promoting physical activity. The rapid uptake of digital platforms, such as social media, short-form video applications, and fitness tracking tools, has significantly altered how students acquire health-related information, monitor their personal behaviors, and engage with feedback mechanisms. These technologies have created new opportunities for accessing sport-related content, raising awareness, and sustaining exercise motivation. However, their fragmented and fast-paced nature may also foster passive consumption, where virtual interaction displaces physical engagement. Meta-analytic findings by Tian et al. further underscore the dual nature of this relationship. On one hand, digital entertainment may crowd out time for physical activity, diminishing participation. On the other hand, when digital media are utilized to support observational learning, motivational priming, and social modeling, they can effectively facilitate greater engagement in sports behavior [7]. This growing tension between online connectivity and embodied activity prompts urgent questions regarding whether and how digital media use translates into meaningful behavioral outcomes [8]. To date, few empirical studies have examined the underlying mechanisms through which digital media use influences sport participation among college students, particularly from cognitive and psychological perspectives. Gaining a deeper understanding of these mechanisms is essential, as behavioral change often depends not only on information exposure but also on individual interpretations, motivational beliefs, and self-regulatory capacity.

This study adopts SCT as its guiding framework to investigate how digital media use shapes sports participation among college students. SCT highlights the reciprocal interaction among environmental factors, cognitive processes, and behavioral outcomes. Within this theoretical structure, the present research explores three interrelated elements: how digital media environments influence students’ physical activity tendencies; how sport cognition serves as a mediating factor that connects media exposure with behavioral decision-making; and how self-efficacy, as a core psychological construct, moderates the relationship between sport cognition and participation by either enhancing or impeding the conversion of intention into action. By clarifying these mechanisms, the study seeks to advance theoretical understanding in the field of sport behavior and to inform the design of more targeted, media-based interventions that support physical activity among university students in a rapidly digitalizing world.

### 1.1. Digital media use and sports participation

Digital media refer to digitally based platforms built upon internet technologies that integrate content creation, dissemination, and interactive functions into a dynamic communication ecosystem. Their open approach to user-generated content, real-time interaction, and capacity to harness collectiveintelligence have fundamentally transformed how information is shared and how social relationships are formed [9]. With the rapid evolution and widespread integration of digital technologies, these media have become an inseparable part of college students’ daily routines [10]. Platforms such as social networking sites, online forums, short video applications, and mobile apps now constitute a diversified media ecology. Building upon the notion that digital media have become deeply embedded in college students’ everyday lives, not only by broadening access to information but also by shaping patterns of social interaction [11], the Uses and Gratifications Theory (UGT) offers a valuable theoretical framework for interpreting the behavioral implications of such media engagement. Rather than passive recipients, individuals are understood as purposive media users who actively select content to satisfy cognitive, emotional, and social needs [12]. In the context of college students, this perspective illuminates how digital platforms are deliberately employed to access exercise-related knowledge, strengthen confidence in performing physical activities, and engage with virtual sport communities that reinforce both identity formation and motivational orientation toward active lifestyles.

However, the influence of digital media is multifaceted and should not be viewed through a purely optimistic lens. While digital platforms offer immediate and abundant access to information, the fragmented nature of content can undermine sustained behavioral commitment. Several studies have indicated that continuous engagement with short video content or live-streamed sports events may lead to a substitution effect, where the consumption of digital content gradually replaces actual physical exercise [8,13,14]. In addition, excessive reliance on health-tracking apps can heighten self-monitoring pressure and performance anxiety, which may ultimately reduce intrinsic motivation and diminish the sustainability of sport participation [15].

Despite these limitations, the potential of digital media to promote physical activity should not be underestimated. SCT offers a comprehensive framework for examining this impact, emphasizing the reciprocal interplay between environmental factors, individual cognition, and behavior [16]. As a powerful environmental driver, digital media provide users with diverse, engaging, and frequently updated sport-related content, as well as virtual platforms for social interaction. These features can serve as external motivators, fostering greater interest and deeper engagement in physical activity among students [7]. A growing body of empirical evidence supports the view that active engagement with sport-related digital content can significantly enhance participation behavior. Students who regularly use media platforms to access updates on athletic events, learn new training methods, or receive health advice demonstrate higher levels of physical activity [17–19]. The increasing availability of sport-related resources through social media and mobile applications has also made it easier for students to acquire knowledge, connect with like-minded peers, and monitor their fitness performance in real time [20]. Particularly noteworthy is the rise of media-based motivational mechanisms, such as community rankings, peer comparisons, and gamified feedback. These not only enhance the visibility and appeal of physical activity but also reinforce its social and interpersonal value. As a result, students are more likely to internalize exercise as a meaningful pursuit and engage in it more consistently [21].

Drawing on the theoretical and empirical foundations discussed above, this study proposes the following hypothesis:

**Hypothesis 1:** The use of digital media positively influences college students’ sports participation.

### 1.2. The mediating role of sports cognition

Sports cognition refers to an individual’s deep understanding and mental processing of sport-related knowledge, value systems, and the perceived significance of physical activity [22]. It plays a central and irreplaceable role in both the initiation and maintenance of sport participation. In the context of digital media, sports cognition does more than shape personal attitudes or general interest in physical activity—it influences how individuals process information and make behavioral decisions based on media input [23]. Although previous studies have addressed factors such as self-regulation [24], perceptual ability [25], and athletic performance, limited attention has been given to the developmental mechanisms of sports cognition among university students in digitally saturated environments. Among college students, the formation of sports cognition has far-reaching implications. A meta-analysis by Keating [26] revealed that sports cognition significantly predicts both the motivation to engage in physical activity and the consistency of such behavior among university students. It not only generates interest in physical activity but also determines how effectively students utilize digital resources for planning and executing their sport-related behaviors. When sports cognition is strengthened, individuals are more likely to sustain performance and participation, particularly in digital contexts where real-time feedback and continuous exposure to information allow for progressive reinforcement of knowledge [27].

The Behavioral Reasoning Theory (BRT) further enriches our understanding of the mediating role of sports cognition by emphasizing the explanatory power of context-specific reasons in bridging values and behavioral intentions. Unlike models that view cognition as a static repository of knowledge, BRT frames cognition as an active evaluative mechanism through which individuals rationalize their actions [23]. In digital environments, students are frequently exposed to sport-related content that aligns with their personal or cultural values, such as health, discipline, and achievement. This exposure typically occurs through platforms that provide real-time feedback, curate content via algorithms, and facilitate validation from peers. These stimuli not only reinforce existing beliefs but also activate internal reasoning that legitimizes the use of digital tools for physical activity engagement. For instance, when students encounter training plans, instructional videos, and performance analytics online, these materials are not passively absorbed; rather, they are cognitively appraised and selectively internalized based on their perceived relevance, credibility, and alignment with individual goals [28]. Through this evaluative process, cognition functions as a gateway that translates media exposure into reasoned behavioral commitment, ultimately reinforcing both the motivation and persistence necessary for sustained participation in physical activity.

Taken together, these findings underscore the mediating function of sports cognition in the relationship between media use and physical activity. Digital media, through rich content presentation and interactive affordances, enhance students’ understanding of sport-specific knowledge, techniques, and strategies. This cognitive engagement goes beyond passive information intake, influencing decision-making processes and strengthening motivational structures [17]. For instance, real-time interactive features provided by digital platforms enable students to build more coherent and personalized fitness routines, thereby fostering greater self-efficacy and behavioral commitment [28]. Empirical evidence provides robust support for this mediating role. Yao et al. [29] found that the internet significantly deepens university students’ understanding of sport-related knowledge, which, in turn, enhances their motivation and actual participation in physical activity. More recently, Shanmugasundaram and Tamilarasu [30] observed that frequent exposure to digital sport content reshapes cognitive structures and fosters more active engagement with information. This heightened cognitive receptivity facilitates both short-term learning and long-term behavioral transformation.

Based on this synthesis of theoretical perspectives and empirical findings, the following hypothesis is proposed:

**Hypothesis 2:** Sports cognition mediates the relationship between digital media use and college students’ sports participation.

### 1.3. The moderating role of self-efficacy

Self-efficacy refers to an individual’s belief in their ability to successfully perform and cope with specific tasks in given contexts. This belief system plays a central role in shaping behavioral choices, the level of effort exerted, the persistence of action, and emotional regulation in the face of challenges [31]. In digital media environments, once students acquire and internalize sport-related knowledge through media exposure, their level of self-efficacy becomes a key determinant in whether this cognition is effectively translated into sustained and consistent physical activity. A substantial body of research has established a strong positive correlation between self-efficacy and sport participation. It is widely recognized as a core psychological mechanism influencing physical activity, motivation, and emotional well-being [32]. For example, Han et al. [33] demonstrated that self-efficacy among college students not only positively predicts their physical fitness and sport performance but also contributes to increased frequency and quality of participation through improved emotional regulation and motivational enhancement. Similarly, Yu et al. [34] found that self-efficacy fosters resilience and the experience of positive emotions, both of which reinforce the sustainability and stability of sports participation.

While the association between self-efficacy and constructs such as resilience [34] and physical health [35] has been extensively explored, relatively few studies have investigated its moderating role in the relationship between sport cognition and behavior. In highly digitalized environments, individuals with strong self-efficacy tend to display greater intrinsic motivation and attentional focus, allowing them to more deeply engage with sport knowledge and more effectively convert that cognition into concrete actions [36]. This effect is particularly salient when students possess a high level of sport cognition but struggle to initiate behavioral follow-through. In such cases, self-efficacy enhances their capacity for self-regulation, enabling them to set realistic physical activity goals and implement personalized strategies for accountability and progress [37]. Moreover, individuals with high self-efficacy are more likely to adopt adaptive coping mechanisms when facing setbacks in sport, favoring emotional regulation and problem-focused strategies over avoidance or disengagement [38]. This proactive coping approach helps preserve behavioral consistency and long-term engagement. Among college students, even those with adequate knowledge about the benefits of physical activity may experience a disconnect between cognition and action if they lack confidence in their ability to perform. In such scenarios, high self-efficacy can serve as a psychological catalyst, strengthening students’ determination to engage in sport, increasing their willingness to face challenges, and ultimately improving both behavioral execution and participation frequency [39].

Based on these theoretical insights and empirical findings, this study proposes the following hypothesis:

**Hypothesis 3:** Self-efficacy moderates the relationship between sport cognition and sports participation among college students.

**Fig 1:**
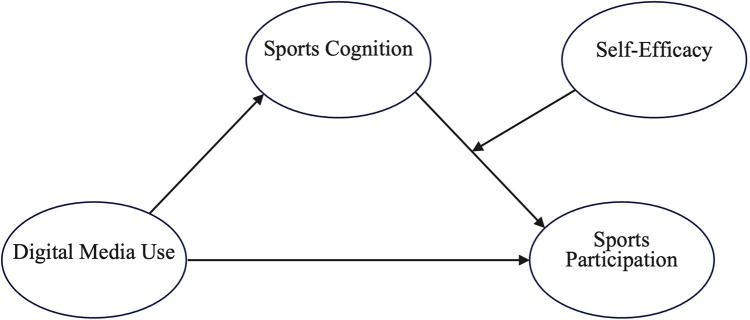
Hypothetical model.

Taken together, this study proposes a moderated mediation model (Fig 1) to examine how digital media use affects students’ sports participation, with sports cognition serving as a mediating mechanism and self-efficacy functioning as a moderator.

## 2. Methods

Although Institutional Review Board (IRB) approval is not mandated in China, we adhered to standard IRB procedures. For instance, no personal identification information was collected. Additionally, a cover letter detailing the study’s purpose and emphasizing the voluntary and confidential nature of participation was provided to all participants. The study protocol was approved by the ethical committee of Jiangxi University of Science and Technology.

### 2.1. Participants and sample size

The participants in this study were full-time undergraduate students drawn from seven comprehensive universities across mainland China, with geographic representation from Beijing, Zhejiang, Jiangxi, and Chongqing. A total of 628 participants were included in the final sample, with ages ranging from 17 to 26 years (M = 20.14, SD = 1.78). Among them, 345 were male (54.9%) and 283 were female (45.1%).

### 2.2. Instruments

#### 2.2.1. Digital media use.

Digital media use was measured using a three-item scale adapted from Xue Tianshan [40], covering three domains: information seeking, knowledge acquisition, and online social interaction. A sample item is: “I often use online platforms to access sport-related knowledge.” All items were rated on a five-point Likert scale (1 = strongly disagree, 5 = strongly agree), with higher scores indicating more frequent digital media engagement. The scale was originally developed within the JSNET2014 framework of the Chinese Social Network and Public Opinion Survey and has demonstrated acceptable psychometric properties. In this study, internal consistency was satisfactory (Cronbach’s α = 0.81) [41]. The composite reliability (CR) was 0.82, exceeding the threshold of 0.70, and above the minimum acceptable level of 0.60 [42]. The average variance extracted (AVE) was 0.61, surpassing the commonly accepted criterion of 0.50 [43], indicating adequate convergent validity (see Table 1).

**Table 1:** Reliability and validity test.

Variable	Item	Cronbach’s α	CR	AVE
Digital Media Use (DMU)	3	0.81	0.82	0.61
Sports Cognition (SC1)	5	0.89	0.89	0.63
Sports Cognition (SC2)	5	0.86	0.87	0.58
Sports Cognition (SC3)	5	0.84	0.87	0.57
Sports Participation (SP)	3	0.82	0.86	0.67
Self-Efficacy (SE1)	10	0.93	0.91	0.52
Self-Efficacy (SE2)	12	0.94	0.93	0.53

#### 2.2.2. Sports participation.

Sports participation was assessed using a three-item scale developed by Man Jianghong [44], capturing exercise frequency, exercise duration, and attendance or viewership of sports events. A sample item reads: “In the past year, how often did you engage in physical exercise during your leisure time?” Responses were rated on a five-point Likert scale (1 = strongly disagree, 5 = strongly agree), with higher scores representing greater participation. This scale has been frequently employed in the Chinese General Social Survey (CGSS) and is recognized for its practical applicability and reliability. In the current study, internal consistency was acceptable (Cronbach’s α = 0.82) [41]. The CR was 0.86, and the AVE reached 0.67, both of which meet the standard thresholds [42,43], suggesting acceptable convergent validity (see Table 1).

#### 2.2.3. Sports cognition.

Sports cognition was measured using a culturally adapted version of the Cognitive Behavioral Physical Activity Scale, originally developed by Schembre et al. [45], and later modified by Eskiler et al. [46] for use among Turkish university students, as referenced by Gülle [47]. The Chinese version was developed via a standard forward–backward translation procedure to ensure conceptual equivalence. The final version used in this study includes 15 items across three subdimensions: outcome expectations, self-regulation, and perceived barriers. Each item was rated on a five-point Likert scale. Higher scores denote stronger cognitive engagement with sport. Internal consistency for the subscales was as follows: outcome expectations (α = 0.89), self-regulation (α = 0.86), and perceived barriers (α = 0.84), all of which met the commonly accepted statistical threshold of 0.70 [41]. The corresponding CR values (0.89, 0.87, and 0.87) and AVE values (0.63, 0.58, and 0.57) also exceeded the commonly recommended benchmarks [42,43], indicating satisfactory composite reliability and convergent validity. (see Table 1).

#### 2.2.4. Self-efficacy.

Self-efficacy was evaluated using the Physical Self-Efficacy Scale developed by Ryckman et al. [48], which comprises 22 items across two subscales: Perceived Physical Ability and Physical Self-Presentation Confidence. All items were rated on a five-point Likert scale, with higher scores reflecting greater perceived physical capability and self-presentation confidence. Internal consistency coefficients for the two subscales were 0.93 and 0.94, both substantially exceeding the conventional reliability benchmark of 0.70 [41], indicating strong internal consistency. CR values were 0.91 and 0.93, surpassing the recommended threshold of 0.70 [42], thereby affirming the scale’s construct reliability. In addition, the AVE values were 0.52 and 0.53, both above the 0.50 criterion [43], suggesting that a sufficient proportion of variance in the observed variables was captured by the latent constructs, thus establishing satisfactory convergent validity within the current sample (see Table 1).

### 2.3. Procedure

Data collection was carried out between September and December 2024, targeting exclusively adult students; no minors were included in the sample. A mixed-mode survey strategy was employed to ensure broad accessibility and enhance response quality. Online questionnaires were distributed via the Wenjuanxing platform, enabling participants to submit responses anonymously through secure digital forms. Simultaneously, offline data collection was organized in classroom settings, where trained members of the research team supervised the administration of identical paper-based surveys to ensure procedural consistency and data integrity across both formats. To enhance both data quality and response rates, the distribution process was standardized across participating universities. University counselors assisted in organizing and disseminating the surveys, while the research team implemented quality control measures such as screening for duplicate IP addresses and identifying abnormal response durations to ensure the authenticity and validity of the collected data. The study adhered to the principles of anonymity and voluntary participation. All respondents voluntarily agreed to participate in the study. All data were used solely for academic research purposes and did not involve the collection or disclosure of any personally identifiable information.

Prior to formal data collection, an a priori power analysis was conducted using G*Power 3.1.9.7 to determine the minimum required sample size. The analysis was based on a linear multiple regression model (fixed model, R^2^ deviation from zero), with a medium effect size (f^2^ = 0.15) as proposed by Cohen [49], a significance level of α = 0.05, a statistical power of 1 − β = 0.95, and four predictors. This threshold, aligned with established conventions in behavioral and social science research [49,50], represents a conservative and widely accepted standard when no strong prior estimates are available [51], ensuring sufficient statistical sensitivity without inflating the sample size unnecessarily. The results indicated that a minimum of 153 participants was required to detect meaningful effects. To account for potential data loss and enhance the robustness of the analysis, a total of 650 questionnaires were distributed. After excluding responses with missing data, logical inconsistencies, or unusually short completion times, 628 valid cases were retained, resulting in a final response rate of 96.61%, well above the required minimum.

### 2.4. Data analysis

All statistical analyses were performed using IBM SPSS Statistics version 26.0 and AMOS version 26.0 (IBM Corp., Armonk, NY, USA), supplemented by the PROCESS macro version 4.2. Confirmatory factor analysis (CFA) was then performed in AMOS to examine the construct validity and overall model fit. Reliability and convergent validity were evaluated via Cronbach’s α, CR, and AVE. Values exceeding 0.70 for α and CR [42], and above 0.50 for AVE [43], were considered indicative of acceptable internal consistency and convergent validity. A range of model fit indices was used to assess the degree of consistency between the model and the observed data. These indices included the chi-square to degrees of freedom ratio (χ²/df), root mean square residual (RMR), goodness-of-fit index (GFI), adjusted goodness-of-fit index (AGFI), normed fit index (NFI), Tucker–Lewis index (TLI), comparative fit index (CFI), root mean square error of approximation (RMSEA), and standardized root mean square residual (SRMR), following established guidelines [52,53]. The results indicated a good model fit, with the following fit statistics: χ²/df = 1.30, RMR = 0.04, GFI = 0.93, AGFI = 0.92, NFI = 0.93, TLI = 0.98, CFI = 0.98, RMSEA = 0.02, and SRMR = 0.03. All values met or exceeded the commonly accepted thresholds for model fit, providing strong evidence for the structural validity of the measurement model (Table 2).

**Table 2:** Model fit indices of the measurement model.

Indicators	χ2/df	RMR	GFI	AGFI	NFI	TLI	CFI	RMSEA	SRMR
Results	1.30	0.04	0.93	0.92	0.93	0.98	0.98	0.02	0.03
Standards	<3.00	<0.08	>0.90	>0.90	>0.90	>0.90	>0.90	<0.08	<0.08
Situation	Fit	Fit	Fit	Fit	Fit	Fit	Fit	Fit	Fit

To address concerns of common method bias (CMB) due to the self-reported nature of the data, Harman’s single-factor test was conducted following the guidelines of Aguirre-Urreta et al. [54]. An exploratory factor analysis extracted seven factors with eigenvalues greater than one, and the first factor accounted for 28.98% of the total variance, which is well below the commonly accepted 40% threshold. This result indicates that common method bias did not significantly compromise the validity of the findings. The data analytic strategy included multiple stages designed to ensure the robustness and validity of the findings. Descriptive statistics and Pearson correlation analyses were conducted in SPSS to explore the basic relationships among the four primary variables: digital media use, sports cognition, self-efficacy, and sports participation behavior.

Subsequent hypothesis testing employed the PROCESS macro in SPSS [55]. Model 4 was used to assess the mediating effect of sports cognition in the relationship between digital media use and sports participation behavior (H1 and H2). Model 14 was applied to test the moderating role of self-efficacy in the pathway from sports cognition to sports participation (H3). A bias-corrected bootstrapping procedure with 5,000 resamples was used to estimate 95% confidence intervals (CIs) for indirect and interaction effects; statistical significance was determined when the CI did not contain zero [56]. To visualize the nature of the moderation, a Johnson-Neyman (J-N) technique was applied to identify the specific range of the moderator for which the conditional effect of the mediator was significant [55]. Additionally, simple slope analyses were conducted to illustrate the direction and strength of the interaction effects [57].

## 3. Results

### 3.1. Correlation analysis of digital media use, sports cognition, self-efficacy, and sports participation

Table 3 presents the means, standard deviations, and Pearson correlation coefficients for all key study variables. Overall, the mean scores of the four variables fall between 3.20 and 3.50 on a 5-point Likert scale, indicating moderate to moderately high levels across the sample. Specifically, digital media use (M = 3.43, SD = 0.87) and sports cognition (M = 3.41, SD = 0.65) showed relatively higher average levels, suggesting that college students in the sample frequently engage with online platforms and possess a generally strong awareness of sport-related concepts. Sports participation (M = 3.50, SD = 0.86) also reflected a moderately high frequency of exercise behavior. In contrast, self-efficacy (M = 3.20, SD = 0.78) showed the lowest mean among the four variables, indicating relatively more variation and potential room for development in students’ confidence toward physical activity.

**Table 3:** Describe statistics and correlation (N = 628).

Variable	M	SD	1	2	3	4
1. Digital media use	3.43	0.87	–			
2. Sports participation	3.50	0.86	0.36**	–		
3. Sports cognition	3.41	0.65	0.49**	0.37**	–	
4. Self-efficacy	3.20	0.78	0.27**	0.25**	0.36**	–

Note: **p < 0.01.

Regarding relationships among the variables, digital media use was significantly and positively correlated with sports cognition (r = 0.49, p < 0.01), suggesting that students who engage more with digital platforms tend to have greater sport-related knowledge and understanding. Sports cognition was positively associated with both sports participation (r = 0.37, p < 0.01) and self-efficacy (r = 0.36, p < 0.01), indicating that cognitive engagement may support both behavioral execution and motivational belief. Additionally, a modest but significant correlation was observed between self-efficacy and sports participation (r = 0.25, p < 0.01). These findings offer preliminary empirical support for the hypothesized mediating and moderating mechanisms, which are examined in detail in the following sections.

### 3.2. Mediating effect of sports cognition between digital media use and sports participation (H1–H2)

To assess whether sports cognition mediates the relationship between digital media use and college students’ sports participation, the analysis was conducted using Model 4 of the PROCESS macro (version 4.2) [55]. As shown in Table 4, digital media use was a significant positive predictor of sports participation (β = 0.37, t = 9.90, p < 0.001), indicating that higher engagement with digital media is associated with more frequent or consistent participation in physical activity. Additionally, digital media use significantly predicted sports cognition (β = 0.36, t = 6.13, p < 0.001), suggesting that digital platforms contribute to shaping students’ understanding and internalization of sport-related knowledge. Moreover, sports cognition itself was a significant predictor of sports participation (β = 0.34, t = 6.11, p < 0.001), reinforcing its role as a potential mediating mechanism.

**Table 4:** Regression results of the mediation model of sports cognition.

Variable	Sports participation		Sports cognition		Sports participation	
	β	t	β	t	β	t
Digital media use	0.37	9.90***	0.36	13.95***	0.24	5.91***
Sports cognition					0.34	6.11***
R^2^	0.139		0.245		0.188	
F	33.69***		67.45***		36.07***	

Note: ***p < 0.001.

To further verify the statistical significance of the mediating pathway, a bias-corrected bootstrapping procedure with 5,000 resamples was employed to estimate the confidence interval of the indirect effect. The results indicated that the indirect effect of digital media use on sports participation through sports cognition was 0.122, with a 95% confidence interval of [0.073, 0.175]. Since the interval did not include zero, the mediating effect was considered statistically significant. Additional calculations revealed that the indirect effect accounted for 33.42% of the total effect, suggesting that sports cognition plays a partial mediating role in the relationship between digital media use and sports participation (Table 5). These findings provide empirical support for both Hypothesis 1 and Hypothesis 2.

**Table 5:** Mediating effect test and 95% CIs of the bootstrap for bias correction.

Effects Type	Paths	Effect Size	Boot SE	95%CL		Proportion
				LLCI	ULCI	
Total effect	Digital media use → Sports participation	0.365	0.037	0.292	0.437	100%
Direct effect	Digital media use → Sports participation	0.243	0.041	0.162	0.323	66.58%
Indirect effect	Digital media use → Sports cognition → Sports participation	0.122	0.026	0.073	0.175	33.42%

Note: The total effect is decomposed into direct and indirect components. Proportions indicate the percentage contribution of each effect to the total.

### 3.3. The moderating role of self-efficacy (H3)

To further investigate whether self-efficacy moderates the mediating pathway between digital media use and sports participation, a moderated mediation analysis was conducted using Model 14 of the PROCESS macro (version 4.2) for SPSS [55]. All continuous variables were mean centered prior to analysis.

The analysis proceeded in two steps. First, the effect of digital media use on sports cognition was tested to establish the mediation path. Second, an interaction term between sports cognition and self-efficacy was added to examine whether it significantly predicted sports participation. A significant interaction would indicate the presence of a moderated mediation effect. The detailed results are presented in Table 6.

**Table 6:** Moderated mediating effect test (n = 628).

Variables	Variable					
	Model 1 (Sports cognition)			Model 2 (Sports participation)		
	β	SE	t	β	SE	t
Digital media use	0.36	0.03	13.95***	0.24	0.04	5.92***
Sports cognition				−0.12	0.15	−0.79
Self-Efficacy				−0.38	0.17	−2.28*
Self-Efficacy * Sports cognition				0.14	0.05	3.03**
R^2^	0.25			0.21		
F	67.45***			27.38***		

Note: ***p < 0.001; **p < 0.01; *p < 0.05.

The regression results revealed that digital media use significantly and positively predicted sports cognition (β = 0.36, t = 13.95, p < 0.001), and sports cognition, in turn, was a significant positive predictor of sports participation (β = 0.24, t = 5.92, p < 0.001). Additionally, self-efficacy not only demonstrated a significant direct effect on sports participation (β = –0.38, t = –2.28, p < 0.05), but its interaction with sports cognition also significantly predicted sports participation (β = 0.14, t = 3.03, p < 0.01). These findings indicate that self-efficacy significantly moderates the effect of sports cognition on sport participation, thereby providing empirical support for Hypothesis 3.

**Fig 2:**
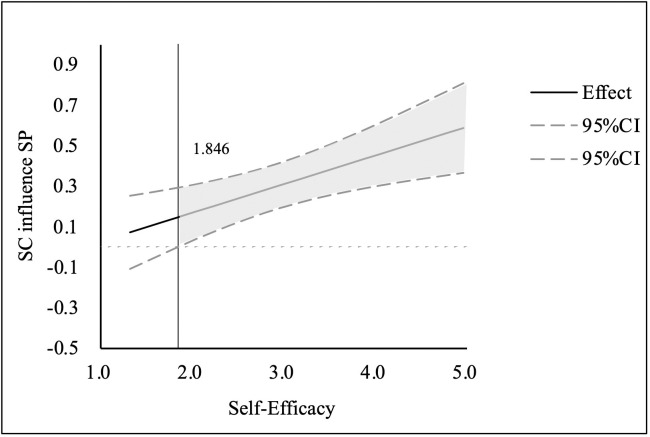
Johnson–Neyman plot. The solid line represents the conditional effect of sports cognition on sports participation at varying levels of self-efficacy. Dashed lines indicate the 95% confidence intervals. The vertical line at 1.846 denotes the threshold above which the effect becomes statistically significant.

To further explore how the moderating effect of self-efficacy varies across different levels, the Johnson–Neyman technique was employed to visualize the conditional effect [57]. As illustrated in Fig 2, the solid line represents the conditional effect of sports cognition on sports participation across the range of self-efficacy, while the dashed lines represent the 95% confidence intervals. The vertical reference line at the value of 1.846 indicates the Johnson–Neyman significance threshold. Specifically, the effect becomes statistically significant (i.e., the confidence interval no longer includes zero) when self-efficacy exceeds this value, marking the beginning of the shaded area where the effect is reliably positive. This finding suggests that the moderating effect is not constant across all levels of self-efficacy. Rather, it becomes more pronounced at moderate to high levels, indicating that strengthening self-efficacy may amplify the positive influence of cognitive factors on behavioral outcomes.

**Fig 3:**
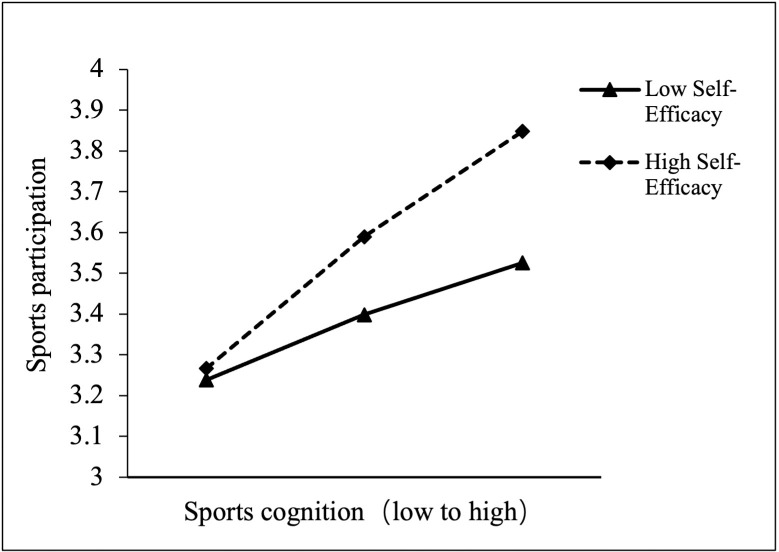
Simple slopes plot. The solid line represents participants with low self-efficacy, while the dashed line represents those with high self-efficacy.

To further elucidate the nature and direction of the interaction effect, a simple slopes analysis was conducted to explore how the relationship between sports cognition and sports participation varies across different levels of self-efficacy. This procedure involved computing the conditional effects of sports cognition at one standard deviation above and below the mean of self-efficacy, thereby allowing for a more granular interpretation of the moderating mechanism. As illustrated in Fig 3, the slope representing high self-efficacy was significantly steeper than that of low self-efficacy, indicating that the positive association between sports cognition and sports participation was markedly stronger among students with higher perceived self-efficacy. Conversely, this relationship was substantially attenuated among individuals with lower self-efficacy.

Finally, a bootstrapping analysis with 5,000 resamples was conducted to examine the indirect effect of digital media use on sports participation through sports cognition at different levels of self-efficacy. The results are presented in Table 7. Among students with high self-efficacy, the indirect effect was 0.18 (SE = 0.03, 95% CI [0.11, 0.24]), whereas for those with low self-efficacy, the indirect effect was 0.09 (SE = 0.03, 95% CI [0.03, 0.15]). In both cases, the CIs did not include zero, indicating that the mediating effect of sports cognition was statistically significant at both levels of self-efficacy. Notably, the indirect effect was stronger in the high self-efficacy group, further supporting the robustness and significance of the moderated mediation model.

**Table 7:** The moderating role of self-efficacy.

Self-Efficacy	Boot Indirect Effect (β)	BootSE	BootLLCI	BootULCI
Low level (M − SD)	0.09	0.03	0.03	0.15
M	0.13	0.03	0.09	0.18
High level (M + 1SD)	0.18	0.03	0.11	0.24

## 4. Discussion

### 4.1. The dual role of digital media: Facilitator and distractor

The findings of this study demonstrate that digital media use serves as a significant facilitator of college students’ engagement in physical activity, aligning closely with the core premise of SCT, which posits that environmental factors shape behavior by enhancing motivational and cognitive processes. In today’s increasingly digitalized ecosystem, sport-related content disseminated via social platforms and mobile applications has not only expanded access to exercise knowledge but also introduced mechanisms that reinforce behavioral commitment [20]. Features such as real-time social comparison, interactive communities, and gamified feedback systems enhance the perceived value and social salience of physical activity, thereby fostering a sustained orientation toward participation [21]. By empirically validating the positive predictive relationship between digital media use and sports participation, the present study substantiates the SCT-informed triadic model of environmental, cognitive, and behavioral interplay within contemporary digital contexts.

However, the role of media is not unilaterally positive. Scholars have increasingly called attention to the potential disruptive functions of media use. While digital platforms offer abundant information and motivation, they may also serve as sources of cognitive interference, leading to attentional fragmentation, misinterpretation of physical activity as passive content consumption, or elevated psychological strain [58]. For instance, while short-form exercise videos on social media may heighten interest in physical activity, they often encourage superficial engagement—replacing actual sport participation with mere viewing behavior [13]. This ambivalence is also evident in the Chinese context. Among college students, excessive reliance on fitness tracking apps has been linked to heightened performance pressure and anxiety, which in turn erodes intrinsic motivation for physical activity [15]. Broader empirical data from the Chinese General Social Survey (CGSS) further reveal that although digital media use is generally associated with increased sports participation, overexposure may shift focus from active engagement to passive consumption. When individuals conflate observation with participation or experience intensified social comparison, media use may paradoxically inhibit rather than promote physical activity [59]. These observations suggest that the positive effects of digital media are conditional and heavily influenced by how, how often, and by whom the media are used.

This duality can be interpreted through multiple theoretical lenses. Information Overload Theory suggests that when individuals are exposed to excessive and rapidly updating content, their cognitive processing capacity becomes diluted, reducing focus and behavioral execution [60]. Meanwhile, Media Dependency Theory [61] emphasizes that the degree of individual dependency on media shapes the way media content is integrated and acted upon. Excessive dependency, particularly when not translated into meaningful behavior, may result in “cognitive substitution” and behavioral disengagement. Such outcomes are especially evident among college students, particularly those with limited self-regulatory capacity or restricted access to physical resources.

Given these insights, both platform developers and university educators must reconsider how to transform media-driven attention into actual behavioral participation. On the one hand, integrating participation-oriented algorithms, such as those that link platform activity with the completion of physical activity tasks, may enhance users’ behavioral responsiveness. On the other hand, universities should encourage hybrid online–offline sport communities that shift students’ roles from passive content recipients to active participants. Moreover, stratified intervention strategies should be developed to address the needs of different user profiles, such as high-frequency immersive users or passive scrollers, in order to prevent the inversion of media’s motivational potential into cognitive distraction. Ultimately, recognizing media as a double-edged sword and managing it accordingly is essential for achieving a healthy and sustainable linkage between information exposure, cognitive engagement, and behavioral action.

### 4.2. Internalization of cognition: Sports cognition as mechanism

Cognition refers to the active mental processing through which individuals interpret and internalize information from their surrounding environments. In the domain of sport behavior, sports cognition extends beyond basic exercise knowledge to include individuals’ evaluative appraisals of physical activity, self-assessments of competence, and expectations regarding outcomes. By transforming external information into internalized motivational drivers, sports cognition functions as a critical intermediary in guiding behavioral decisions. Findings from the present study indicate that the effect of digital media on students’ sport participation is not direct, but rather channeled through sports cognition. This underscores the importance of cognitive internalization: the sport-related content encountered through digital media is not simply received but cognitively processed and assimilated into one’s mental framework. Through this process, students develop positive expectations and enhanced self-efficacy, which in turn promote behavioral engagement and persistence. This cognitive pathway is consistent with the environment–cognition–behavior sequence posited by SCT, and it also parallels the attitude–intention–behavior chain central to the Theory of Planned Behavior (TPB) [62], wherein belief-based attitudes serve as proximal determinants of behavioral intentions and eventual actions. Specifically, the findings illustrate that digital media do not exert uniform effects on behavior; rather, their influence is contingent upon users’ cognitive appraisals and motivational interpretations. This process also reflects the core assumptions of Expectancy–Value Theory. When individuals’ sport cognition includes a clear appraisal of expected benefits (e.g., health improvement, social connection), a realistic assessment of feasibility (e.g., perceived competence, accessible environment), and a willingness to self-regulate (e.g., setting goals, tracking feedback), they are more likely to transition from knowing what is useful to acting on it [63]. In the context of university students, digital media—particularly those offering interactive features and immediate feedback—play an instrumental role in strengthening self-efficacy, clarifying outcome expectations, and enhancing goal salience. These mechanisms collectively facilitate the conversion of externally provided cues into internally regulated and autonomous engagement in physical activity [64].

However, the mediating role of sports cognition may not function uniformly across all contexts. On one hand, media environments saturated with fragmented and fast-paced content can inhibit deep cognitive engagement, leaving individuals with only surface-level impressions of sport and limiting the development of intrinsic motivation [65,66]. On the other hand, cognitive conversion is contingent on several internal resources, such as cognitive capacity, media literacy, and self-efficacy. For students with underdeveloped cognitive structures or limited digital competence, frequent exposure to external content may fail to foster a stable understanding of sport, thereby weakening the mediating role of cognition.

Considering these challenges, both educators and media platform designers must consider strategic interventions aimed at enhancing cognitive integration. Efforts should be directed at three key areas: content adaptation, cognitive scaffolding, and feedback guidance. First, streamlined and well-structured information delivery can reduce cognitive load and improve depth of understanding. Second, narrative formats, situational simulations, and role-model-based content can promote emotional resonance and internalization. Third, integrated systems that link cognition and behavior, such as closed-loop mechanisms that connect learning, practice, and feedback, can strengthen the translation of knowledge into action. Through such mechanisms, sports cognition can serve its intended role as a psychological bridge, linking media exposure to sustained behavioral engagement.

### 4.3. Strengthening conversion: The regulatory role of self-efficacy

Self-efficacy, defined as an individual’s belief in their ability to successfully carry out a specific behavior, functions as a critical psychological mechanism in determining whether cognition is effectively translated into action. Within the context of sport behavior, self-efficacy not only influences whether individuals believe they can participate, but also governs their willingness to persist in the face of obstacles, pressure, or limited feedback [16]. The present study revealed a significant moderating effect of self-efficacy on the relationship between sports cognition and sports participation: the stronger the individual’s self-efficacy, the more likely cognitive understanding was to lead to behavioral engagement. This underscores the path-enhancing function of self-efficacy as a psychological resource.

Participants with high self-efficacy in this study were more capable of interpreting their sport-related knowledge as actionable plans and mobilizing the necessary behavioral resources to carry them out. This finding aligns closely with the “perceived behavioral control” component of the TPB [62]. Moreover, the Control–Value Theory [67] also highlights that individuals are more likely to internalize sport participation intentions when they both value the activity and believe they possess the ability to perform it successfully. Together, these theories illustrate the essential role of self-efficacy as a environment-cognitive-behavior bridge that enables the transformation of internal beliefs into external behavior.

It is important to note, however, that the moderating role of self-efficacy is bounded by contextual and individual factors. On one hand, self-efficacy is heavily shaped by prior success experiences and positive feedback. If an individual has repeatedly experienced failure in previous sport activities, their self-efficacy, even when externally stimulated, may remain fragile or unstable [68]. On the other hand, although digital media provide instant feedback and social reinforcement, their fragmented content and comparative social dynamics may lead to inflated self-perceptions or increased performance pressure. For students with low psychological resilience or limited self-regulatory capacity, this can result in motivational depletion rather than empowerment [69]. These findings suggest that the positive moderating effect of self-efficacy is not universal. It is more likely to remain stable and effective among individuals with more mature cognitive structures and stronger self-regulation skills.

In light of these mechanisms and boundary conditions, multi-level intervention strategies are essential to activate and sustain the functional value of self-efficacy. At the platform level, designers should incorporate phased, visible goals, real-time positive feedback systems, and user growth curves to help individuals derive a sense of genuine competence through the completion of small, attainable actions. At the educational level, institutions could implement tiered participation tasks, self-assessment tools for sport learning, and feedback systems involving instructors and peers to help students build both confidence and behavioral habits. At the individual level, interventions should focus on strengthening metacognitive skills and action planning strategies. For example, students could be guided to break down sport goals into smaller components, track their behavioral progress, and engage in self-reinforcement to strengthen the cognition-to-action pathway.

Only through the coordinated functioning of these interventions can self-efficacy truly act as a behavioral catalyst by amplifying the motivational energy that transforms sport cognition into sustained sport participation. This process is essential for enabling students to move from simply understanding the importance of physical activity to actively engaging in it on a consistent basis.

### 4.4. Limitations and contributions

While this study offers meaningful insights into the psychological mechanisms linking digital media use and sports participation among college students, several methodological limitations warrant further consideration. First, the cross-sectional nature of the data collection limits the ability to draw causal inferences. This design captures only a snapshot of participant behavior at a single time point, making it difficult to ascertain the temporal order of variables. Second, reliance on self-reported data introduces potential biases that may undermine measurement accuracy. Participants may overestimate or underestimate their actual behavior due to recall errors, social desirability tendencies, or subjective interpretation of survey items. These systematic biases can distort observed relationships, particularly when assessing sensitive or evaluative constructs such as self-efficacy or exercise frequency. Such distortions complicate the interpretation of effect sizes and may mask or exaggerate the true underlying associations. Third, although the sample was randomly drawn from comprehensive universities, the study did not control for students’ academic major, particularly those enrolled in sports science programs. Individuals with formal training in sport-related disciplines may possess systematically different levels of sports cognition, motivation, and behavioral patterns, thereby introducing potential confounds into the observed associations. Future research should stratify samples based on disciplinary background or include major as a covariate in statistical models to improve internal validity.

Despite these limitations, the study offers significant theoretical and practical contributions. From a theoretical standpoint, it integrates SCT with Uses and Gratifications Theory, proposing a novel framework that traces the pathway from information input to cognitive processing, efficacy modulation, and behavioral output. This interdisciplinary approach extends the conceptual landscape of media psychology and sport behavior research. Empirically, the study adopted a rigorous moderated mediation analysis to simultaneously test the mediating effect of sports cognition and the moderating effect of self-efficacy, contributing robust evidence to our understanding of youth sport participation in digital contexts. Practically, the findings offer concrete insights for designing college sport programs, enhancing digital health platforms, and informing public health policy. Universities may leverage digital media to guide students’ cognitive and motivational engagement with sport, digital platforms can optimize content to foster positive user experiences, and policymakers may draw on these mechanisms to develop more targeted strategies for promoting physical activity among young adults.

## 5. Conclusions

While previous studies have established a link between media use and physical activity [59], this study advances the literature by unpacking the mediating and moderating mechanisms within the Chinese university context. Specifically, the findings demonstrate that digital media use indirectly facilitates sports participation among college students by enhancing their sports cognition. Self-efficacy further serves as a critical moderator, strengthening the translation of cognitive understanding into sustained behavioral engagement. These results contribute to a more nuanced integration of SCT and media-use frameworks in the domain of sport behavior, highlighting the interplay between environmental inputs, individual cognition, and motivational dynamics. At the same time, they also validate the applicability of the TPB framework in this context, affirming that attitudes shaped by cognitive evaluations can lead to behavioral intentions and ultimately foster physical activity engagement. Moreover, the study offers valuable theoretical refinement by incorporating cognitive appraisal and self-efficacy as key mechanisms within behavioral models. From a practical standpoint, the insights gained underscore the need for targeted interventions in university-based physical activity programs and support the strategic design of digital platforms aimed at fostering intrinsic motivation and health-oriented behavior change among emerging adults.

## Supporting information

S1 File Supplementary materials. Raw data and all statistical procedures used in the present study.
(RAR)
